# Bile resistance mechanisms in *Lactobacillus* and *Bifidobacterium*

**DOI:** 10.3389/fmicb.2013.00396

**Published:** 2013-12-24

**Authors:** Lorena Ruiz, Abelardo Margolles, Borja Sánchez

**Affiliations:** Laboratory of Probiotics and Prebiotics, Department of Microbiology and Biochemistry of Dairy Products, Instituto de Productos Lácteos de Asturias – Consejo Superior de Investigaciones CientíficasAsturias, Spain

**Keywords:** *Lactobacillus*, *Bifidobacterium*, bile resistance, bile adaptation, bile detoxification

## Abstract

Probiotics are live microorganisms which when administered in adequate amounts confer a health benefit on the host. Most of the probiotic bacteria currently available in the market belong to the genera *Lactobacillus* and *Bifidobacterium*, and specific health-promoting activities, such as treatment of diarrhea or amelioration of gastrointestinal discomfort, have been attributed to them. In order to be able to survive the gastrointestinal transit and transiently colonize our gut, these bacteria must be able to counteract the deleterious action of bile salts, which are the main components of bile. Bile salts are detergent-like biological substances synthesized in the liver from cholesterol. Host enzymes conjugate the newly synthesized free bile acids in the liver with the amino acids glycine or taurine, generating conjugated bile salts. These compounds are stored in the gall bladder and they are released into the duodenum during digestion to perform their physiological function, which is the solubilization of fat coming from diet. These bile salts possess strong antimicrobial activity, since they are able to disorganize the structure of the cell membrane, as well as trigger DNA damage. This means that bacteria inhabiting our intestinal tract must have intrinsic resistance mechanisms to cope with bile salts. To do that, *Lactobacillus* and *Bifidobacterium* display a variety of proteins devoted to the efflux of bile salts or protons, to modify sugar metabolism or to prevent protein misfolding. In this manuscript, we review and discuss specific bile resistance mechanisms, as well as the processes responsible for the adaptation of bifidobacteria and lactobacilli to bile.

## INTRODUCTION

Strains of *Lactobacillus* and *Bifidobacterium* have been extensively used as probiotic microorganisms for humans (Sánchez et al., 2012). In order to reach the colon in a viable state, they must cope with specific stress challenges throughout the gastrointestinal tract, among which the presence of bile in the upper parts of the small intestine is one of the main ones. The main components of bile are bile acids, which are produced and conjugated with the amino acids glycine or taurine in the liver, to generate conjugated bile salts (Hofmann, 1994). Bile is stored in the gall bladder and flows from there to the duodenum during digestion, facilitating the solubilization and absorption of dietary fats. Thus, under normal physiological conditions, our intestine holds a bile salt concentration gradient ranging from more than 40 mM to less than 1 mM – equivalent to a range between 2% and 0.05% – which is responsible, among other factors, for shaping the microbial community profile found in our gut (Islam et al., 2011).

Apart from its normal physiological function, bile is highly toxic for those microorganisms unadapted to the intestinal conditions. Therefore, enteric bacteria, including lactobacilli and bifidobacteria, must have evolved specific defense mechanisms to resist the deleterious action caused by these compounds. The strong lipophilic nature of the steroid ring makes the cell membrane the main target of these molecules, in which they disturb the lipid packaging and disrupt the proton motive force, causing cell death (Kurdi et al., 2006). Furthermore, since the unconjugated forms are weak acids, they can passively diffuse into the cell and, once inside, they are dissociated producing an acidification of the cytoplasm (Sánchez et al., 2013). Other side effects induced by bile have been documented, including induction of oxidative stress and DNA repair mechanisms, alterations of sugar metabolism, and protein misfolding (Begley et al., 2005). Thus, in this review we would like to summarize the current knowledge on the mechanisms used by lactobacilli and bifidobacteria to counteract the effect of bile acids on cell physiology.

## COMMON ASPECTS OF BILE RESISTANCE MECHANISMS IN *Lactobacillus* AND *Bifidobacterium*

**FIGURE 1:**
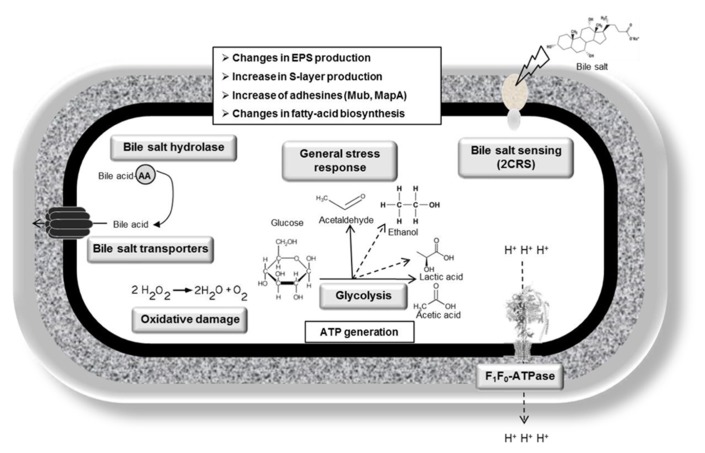
**Bile response mechanisms identified and characterized in lactobacilli**.

Bile tolerance is one of the most crucial properties for probiotic bacteria, as it determines its ability to survive in the small intestine, and consequently its capacity to play its functional role as a probiotic. Although intrinsic bile tolerance appear to be strain-dependent, both lactobacilli and bifidobacteria can progressively adapt to the presence of bile salts, and resistant derivatives can be obtained from sensitive wild type strains by subculturing in gradually increasing concentrations of bile (Noriega et al., 2004; Guglielmotti et al., 2007; Burns et al., 2010). On some occasions, bile salt-resistant strains can also be obtained by selection toward other stress conditions, such as acid pH (Chou and Weimer, 1999); and bile-adapted strains usually display cross-resistances to other stress factors (Margolles et al., 2003). Indeed, this reflects the existence of common mechanisms in bacterial responses to various stresses and suggests that enhancing probiotics bile tolerance could help to develop more robust strains displaying enhanced resistance to other technological or gastrointestinal factors compromising probiotics survival (Sánchez et al., 2012). Bile-adapted strains also provide an interesting model to analyze the molecular mechanisms involved in bacterial tolerance and response to these compounds. Indeed, by using high-throughput techniques on some of these bacterial models some pivotal aspects mediating bile resistance and response in these microorganisms have been identified (Sánchez et al., 2007b; Burns et al., 2010). Overall, bile response is a multifactorial phenomenon, implicating a variety of processes addressed toward detoxification of bile and counteracting the deleterious effect on bacterial structures, as described on the following paragraphs. Active efflux of bile acids/salts (Pfeiler and Klaenhammer, 2009; Bustos et al., 2011; Ruiz et al., 2012a,b), bile salt hydrolysis (Kumar et al., 2006; Lambert et al., 2008), and changes in the architecture/composition of cell membrane and cell wall (Gómez-Zavaglia et al., 2002; Taranto et al., 2003; Ruiz et al., 2007) appear to be the most prevalent bile-specific mechanisms mediating resistance in both genera. In addition, general stress response, protection against oxidative damages, as well as global glycolytic reorganizations are other common consequences of bile exposure, that might be employed to counteract some of the cellular damage caused by these compounds (**Figure 1**; **Table 1**; Hamon et al., 2011; Ruiz et al., 2011; Alcantara and Zuñiga, 2012).

**Table 1:** Strategies and molecular elements involved in bifidobacterial bile response and adaptation. Bile induced (+); bile repressed (-).

Strategy	Molecular mechanisms and players	Species	Reference
**Bile salt deconjugation (unclear)**
	*bsh*	*B. animalis*	Kim and Lee (2008)
**Bile efflux**
	*Bbr_0838* (+)	*B. breve*	Ruiz etal. (2012a, b)
	*ctr* (constitutive)**	*B. longum*	Price etal. (2006)
	*betA* (+)**	*B. longum*	Gueimonde etal. (2009)
**Counteracting H+ accumulation**
	F_0_F_1_-ATPase (+)	*B. animalis*	Sánchez etal. (2006)
**General stress response**
	HtrA, DnaK, GroEL	*B. animalis and B. longum*	Sánchez etal. (2008)
Counteracting redox state	Methionine synthase, peroxidase	*B. longum*	Sánchez etal. (2007a)
**Surface properties**
Surface proteome	DnaK (+) – (colonization factor?)	*B. animalis*	Candela etal. (2010)
	Enolase (+) (colonization factor?)	*B. longum*	Ruiz etal. (2009)
	OppA (+)	*B. longum*	Ruiz etal. (2009)
EPS	*p-gtf* (+)	*B. animalis*	Ruas-Madiedo etal. (2009)
	EPS production related to bile tolerance	*B. breve*	Fanning etal. (2012)
	EPS production related to bile tolerance	*B. breve, B. bifidum and B. pseudocatenulatum*	Alp and Aslim (2010)
Fatty acids	Bile response and adaptation related to changes in fatty acids composition	*B. animalis*	Ruiz etal. (2007)
	Bile response related to changes in fatty acids composition	*B. bifidum and B. pseudolongum*	Gómez-Zavaglia et al. (2002))
**Carbon metabolism**
Fluctuation fermentable carbon sources	Glycosidases	*B. animalis*	Ruas-Madiedo etal. (2005)/Noriega etal. (2004)
Increase ATP production	Glycolitic enzymes (+)	*B. longum*	Sánchez etal. (2005)
	F6PPK/GA3PDH (+)	*B. animalis*	Sánchez etal. (2007b)
**Others**
	ABC-type and MDR transporters	*B. breve*	Ruiz etal. (2012b)

### ROLE OF BILE-EFFLUX SYSTEMS

The active extrusion of the bile acids and salts that accumulate in the cytoplasm through efflux pumps is a common bacterial mechanism to counteract bile toxicity (Piddock, 2006). To date, a number of multidrug transporters (MDRs) belonging to the ATP-binding cassette or the major facilitator superfamily, have been described to mediate bile tolerance in lactobacilli and bifidobacterial strains: four transporters in *Lactobacillus acidophilus* NCFM, LBA0552, LBA1429, LBA1446, and LBA1679 (Pfeiler and Klaenhammer, 2009); one in *Lactobacillus reuteri* ATCC 55730, *lr1584* (Whitehead et al., 2008); two in *Bifidobacterium longum*, *ctr* and *BL0920* (Price et al., 2006; Gueimonde et al., 2009), and one in *Bifidobacterium breve*, *Bbr_0838* (Ruiz et al., 2012a,b). Indeed, deletion of any one of LBA0552, LBA1429, LBA1446, and LBA1679 transporters in the strain *L. acidophilus* NCFM rendered the mutant strains more sensitive to bile and certain antibiotics (Pfeiler and Klaenhammer, 2009); and mutation of *lr1584* in *L. reuteri* reduced the strain capability to grow in the presence of bile and completely abolished its capacity to acquire bile-tolerant phenotypes (Whitehead et al., 2008). In bifidobacteria, only *Bbr_0838* has been inactivated, through insertional mutation, a change that reduced the strain’s capability to grow in the presence of cholic acid (Ruiz et al., 2012a). Previous results on the BL0920 transporter from *B. longum*, which shares a high degree of homology with *Bbr_0838,* also suggest a role in bile protection (Gueimonde et al., 2009; Ruiz et al., 2012b).

The active extrusion of labeled bile has been demonstrated for the bifidobacterial transporters BL0920 and ctr (Price et al., 2006; Gueimonde et al., 2009). In *Lactobacillus johnsonii*, a functional taurocholic and cholic acid antiporter belonging to the major facilitator superfamily, CbsT2*,* has also been described although its contribution to bile tolerance has not been demonstrated by means of functional genetics (Elkins and Savage, 2003). Furthermore, *L. reuteri* efflux of both free and conjugated bile acids has been demonstrated and ATP was found to be the main force driving the extrusion activity (Bustos et al., 2011).

Remarkably, all transporters identified to date in lactobacilli and bifidobacteria mediating bile-tolerance and/or extrusion activity, exhibit some degree of bile-inducibility (Gueimonde et al., 2009; Koskenniemi et al., 2011; Ruiz et al., 2012b). The bifidobacterial genes *BL0920* and *Bbr_0838*, exhibited the highest levels of transcriptional induction following bile exposure (Gueimonde et al., 2009; Ruiz et al., 2012b) and appear to present homologs in bifidobacterial strains of intestinal origin (Gueimonde et al., 2009). Preliminary characterization of their promoter regions identified putative regulatory elements; however, specific transcriptional regulators have yet to be identified. This will significantly contribute to our understanding on the acquisition and evolution of traits conferring a selective advantage within the intestinal environment.

### BILE-SALT HYDROLASES

Among the different mechanisms deployed by bacteria to counteract the harmful effect of bile, the activity of bile-salt hydrolases (BSHs) has been proposed to confer protection through bile salt deconjugation. BSHs belong to the chologlycine hydrolase family of enzymes, and have been proposed to have evolved as an adaptation to bile-containing environments (Begley et al., 2005; Jones et al., 2008). BSH catalyzes a reaction in which glycine and taurine are de-conjugated from bile salts, and the corresponding unconjugated acids can be further metabolized by other gut bacteria (De Boever et al., 2000). In *Lactobacillus amylovorus* and *Lactobacillus plantarum*, a comparison between wild type and mutated BSH established a link between BSH activity and bile tolerance (Begley et al., 2005). Such comparisons have not been performed on bifidobacterial BSH, however compiling evidence suggest a role of the enzyme in bifidobacterial bile resistance. For instance, BSH appear over-represented in a bile-adapted *Bifidobacterium animalis* strain that also displays higher hydrolyzing activity than its wild type counterpart (Noriega et al., 2006; Sánchez et al., 2007b). Nevertheless, the mechanism by which BSH may confer bile protection is not fully understood since unconjugated forms are more hydrophobic and toxic as they can freely enter the cells, so they need to be actively pumped outside. However, they are weaker acids than their conjugated counterparts, thus recapturing the co-transported proton may counteract the drop in pH that take place in bile environments (Begley et al., 2005). Remarkably, BSH homologs are only present in bile containing environments, reflecting its importance to enhance bacterial competitiveness within the intestine (Jones et al., 2008). However, there is no agreement on its significance for *in vivo* persistence of lactobacilli and bifidobacteria, although this role has been unequivocally proven in other microorganisms like *Listeria* spp. (Kumar et al., 2012).

Bile-salt hydrolase is an inducible activity in *Lactobacillus*, and expression of *bsh* gene in *L. plantarum* was increased sixfold after exposure to 2% bile (Duary et al., 2012). *In vitro* experiments suggested the activity is constitutively expressed in bifidobacteria (Sánchez et al., 2005), although *in vivo* assays revealed intracellular accumulation of this enzyme in *B. longum*, in the gut of rabbits. This pointed to intestinal factors, other than bile, triggering its expression (Yuan et al., 2008) and supports the significance of this activity within the intestinal environment. Lactobacilli and bifidobacteria can harbor several functional copies of BSH genes within their genomes, all of them participating in bile salt deconjugation, with a substrate preference (Ren et al., 2011). Remarkably, BSH specificity seems to rely on the specific amino acid and hence, BSH has also been proposed to confer a nutritional advantage on producing bacteria, through capturing the amino acid moieties released from its hydrolyzing activity (Begley et al., 2005). Interestingly, BSH and bile salt transporters are sometimes found organized in operons, notably in lactobacilli strains isolated from the human environment and not from dairy products (Elkins et al., 2001; Elkins and Savage, 2003).

Gut microbiota BSH activity has also been related to effects on the host. Increases in the BSH levels have been linked to a higher cholesterol-removing capacity, which may be considered beneficial for the human host (Dong et al., 2012). However, unconjugated bile acids are not as well re-absorbed and can be further transformed into secondary bile acids whose accumulation in the colon has been speculated to cause certain tissue damage (Li and Chiang, 2012). Overall, evidence suggests that BSH enzymes play a significant role for gut bacteria, presumably contributing to bile tolerance, although the mechanisms are not completely understood. Similarly, the impact of this gut-microbiota encoded activity on the host needs further investigation.

### EFFECTS OF BILE SALTS ON THE BACTERIAL ENVELOPES AND FATTY ACID METABOLISM

Due to its lipophilic character, bacterial membranes represent one of the main targets of bile that disrupts the structure of bacterial envelopes, affecting both cell and colony morphology (Suskovic et al., 2000; Margolles et al., 2003; Kurdi et al., 2006). This effect has been evaluated and used as a bile salt-resistance marker in certain *Lactobacillus* strains, since rough colonies are more sensitive than smooth colonies, probably in connection to changes in envelope architecture (Suskovic et al., 2000). Furthermore, changes in the lipid composition of bacterial membranes have been described following bile exposure in bifidobacteria and lactobacilli (Gómez-Zavaglia et al., 2002; Kociubinski et al., 2002; Taranto et al., 2003; Ruiz et al., 2007). Remarkably, in *B. animalis* IPLA4549 bile has been suggested to promote changes in the composition of the membrane lipids through changes in the production of proteins involved in fatty acid metabolism (Sánchez et al., 2007b). These observations correlate to transcriptomic data of bile-exposed *B. animalis* BB12, and are consistent with studies that demonstrate bile-induced changes in bifidobacterial membrane composition and surface properties (Gómez-Zavaglia et al., 2002; Kociubinski et al., 2002; Savijoki et al., 2005; Ruiz et al., 2007). Similarly, fatty acid changes described in lactobacilli following bile exposure (Taranto et al., 2003) are in agreement with variations in enzymes involved in fatty acid metabolism, as revealed through proteomic and transcriptomic approaches in *Lactobacillus rhamnosus* GG (Koskenniemi et al., 2011). However, these changes appear to be strain-dependent and therefore it is difficult to interpret how they contribute to the defense against bile toxicity. They have been proposed to result in alterations in the physicochemical properties of the membranes and cell wall functionalities which might contribute to reduce bile diffusion (Gómez-Zavaglia et al., 2002; Taranto et al., 2006; Ruiz et al., 2007). For instance, bile exposure in bifidobacteria was associated to increased hydrophobicity and reduced z-potential, due to the bile moieties accumulated within the membranes (Kociubinski et al., 2002). In *B. animalis* IPLA4549 bile adaptation also resulted in a strain with a higher proportion of saturated fatty acids, and displaying reduced membrane fluidity (Ruiz et al., 2007).

Other genes coding for surface-associated proteins, such as mucus-binding protein (*mub*), or mucus adhesion promoting protein (*mapA*) in *L. plantarum* isolates, appeared over-represented when the growing media was supplemented with a mix of mucin (0.05%) and bile (1%; Duary et al., 2012). Similarly, in a *B. longum* strain, surface associated enolase and DnaK, which are able to capture human plasminogen, appeared up-regulated by bile, thus suggesting that bile modulates molecular traits exerting a role in intestinal colonization (Candela et al., 2009, 2010; Ruiz et al., 2009). However, these results could not always be correlated with increased adhesion to intestinal cell lines or mucin. For instance, a bile salt-adapted strain of *Lactobacillus delbrueckii* subsp. *lactis* showed reductions in cell hydrophobicity, auto-aggregation, and adhesion to human cell lines, despite improved resistance to physiological bile-salt concentrations (Burns et al., 2011a). On the contrary, bile adaptation in *B. animalis*, *B. longum*, and *Bifidobacterium bifidum* strains was correlated with increased adhesion to intestinal mucus *in vitro* and, although the presence of physiological concentrations of bile reduced the adhesion in all cases, the adapted strains still displayed higher binding capacity than their original counterparts (Gueimonde et al., 2007). However, the effect of a simulated gastric transit on pairs of *B. animalis* and *B. longum* strains, including the wild type and their bile-adapted counterparts, showed no improved adherence of bile-adapted strains to intestinal cell lines *in vitro* (de los Reyes-Gavilán et al., 2011). Therefore, further research is needed to confirm whether *in vitro* bile adaptation improves *in vivo* performance.

Production of external exopolysacharide (EPS) layers is an extended trait among intestinal bacteria (Ruas-Madiedo et al., 2007). These exocellular polymers cause a deep impact on bacterial surface properties and act as a protective coat against environmental conditions (Alp and Aslim, 2010; Leivers et al., 2011; Fanning et al., 2012). In accordance with this, bile has been demonstrated to induce exopolysaccharide production in *B. animalis* IPLA4549, probably as a mechanism of bile protection (Ruas-Madiedo et al., 2009). In fact, *in vitro* and *in vivo* models revealed a correlation between EPS production and bile tolerance in other bifidobacteria. For instance, in *B. breve* UCC2003, the EPS coat was essential for bile survival and *in vivo* colonization of the mice gut (Fanning et al., 2012). Nevertheless, the effect of bile on EPS production in lactobacilli is not as clear. Whereas transcriptomic and proteomic data in *L. rhamnosus* GG point to a reduced production of enzymes involved in EPS biosynthesis in bile-containing environments (Koskenniemi et al., 2011) in *L. delbrueckii* no variations were found following bile exposure, although acquisition of stable bile-resistance was correlated to a significant overproduction of enzymes involved in EPS biosynthesis (Burns et al., 2010). It still remains to be determined whether bile exposure affects the composition and properties of the EPS layers. Finally, other cell-wall structures may be affected in response to bile, as in the case of *L. acidophilus*, which increases S-layer protein production at genetic level when cultured in the presence of 0.05% bile (Khaleghi et al., 2010).

Therefore, *in vitro* analyses show that bile deeply affects surface properties of lactobacilli and bifidobacteria, due to changes on cell wall architecture, lipid composition, presence and characteristics of external coats. Some of these changes have been determined to occur at transcriptional level and may affect bacterial capability to interact with the intestinal epithelia. It still remains to be determined whether bile-acquired tolerance and bile regulation of putative colonization factors translate into better *in vivo* probiotic behavior.

### GENERAL STRESS RESPONSE

It is known that, in addition to their action as detergents, bile salts impose oxidative stress on bacteria, due to the production of reactive oxygen/nitrogen species (Sokol et al., 1993; Bernstein et al., 1999; Begley et al., 2005). In addition, bile salts deconjugation releases protons, thus causing an intracellular acidification (Begley et al., 2005). Accordingly, some of the pathways activated in bacteria following a bile challenge are those related with general, acid and oxidative stress responses, as revealed by microarray experiments in *L. reuteri, L. rhamnosus, L. plantarum, L. johnsonii,* and *B. breve* (Bron et al., 2004, 2006; Whitehead et al., 2008; Koskenniemi et al., 2011; Ruiz et al., 2012b; Lee et al., 2013), but also by various-omic approaches in other enterobacteria such as *Enterococcus faecalis* (Rincé et al.,2003) or *Salmonella enterica* (Hernández et al., 2012), among others. The aim of this response is to counteract the negative effects of bile at the level of cell wall disorganization, oxidative stress and DNA damage/protein denaturation and intracellular acidification. In fact, bile exposed bacteria overexpress a range of proteins to counteract these effects. Damage to proteins is counteracted through a chaperone/protease mediated response which promotes a quick recycling of damaged proteins and a proper folding of nascent proteins. In bifidobacteria, overproduction of a battery of proteases and chaperones upon either bile response or adaptation has been shown (Sánchez et al., 2005, 2007b, 2008; Savijoki et al., 2005). The range of bile-induced chaperones/proteases was broader in *B. animalis* than in *B. longum*, with three main chaperones common to both species, HtrA, GroEL, and DnaK, the latter also having been implicated in ox-gall adaptation in *Bifidobacterium adolescentis* NCC251 (Schmidt and Zink, 2000). Some chaperones, ClpP, Dps, GroEL, Hsp1, and Hsp3, were also found to be up-regulated in *L. plantarum* (Hamon et al., 2011). In agreement with this, mutations in the Clp chaperone in *L. reuteri* were also associated with reduced survival in the presence of bile (Whitehead et al., 2008). In the case of *L. acidophilus*, a decrease in H_2_O_2_ formation was also observed after treatment with 0.1% bile and, although the molecular mechanisms responsible of this effect have not been discerned yet, this suggests that activities aimed at reducing production of oxidant molecules could enhance bile tolerance (Khaleghi et al., 2010). In fact, a DPS protein (DPS: DNA-binding protein from starved cells) and a thioredoxin-dependent thiol peroxidase, both involved in SOS response, are overproduced in a bile-exposed *B. animalis* subsp. *lactis* strain (Sánchez et al., 2007b). Also, co-expression of catalase gene *katA* from *Lactobacillus sakei* and the bile salt hydrolase gene *bsh1* from *L. plantarum* in *Lactobacillus casei* HX01, resulted in higher resistance to both oxidative and bile salt stress (Wang et al., 2011). Furthermore, the F_0_F_1_ATPase responsible of ATP generation while pumping protons outside the cells, has been described as the molecular link connecting both acid and bile stress responses in *B. animalis* (Sánchez et al., 2006). In fact, F_0_F_1_ATPase has been found to be up-regulated under bile environments in a variety of bacteria (Hamon et al., 2011; Koskenniemi et al., 2011) and seems to play a crucial role in maintaining the intracellular pH under bile environments.

Two-component regulatory systems (2CRS) have been implicated in sensing bile salt presence in *L. acidophilus* (Pfeiler et al., 2007). An operon encoding a 2CRS, a transporter, an oxidoreductase and four hypothetical proteins was shown to be over-expressed as a response to bile in *L. acidophilus* by transcriptomics. Interestingly, mutations in the genes coding for the 2CRS, in the transporter and in one of the hypothetical proteins resulted in lower bile salt resistance, while mutations in the oxidoreductase and in another hypothetical protein induced an increase in bile salt tolerance (Pfeiler et al., 2007). The involvement of 2CRS in sensing bile salts has also been described in enterobacteria (Kus et al., 2011). In bifidobacteria, no bile-sensing systems or specifically bile controlled transcriptional regulators have been identified yet.

### CHANGES IN CENTRAL METABOLIC PATHWAYS. A FOCUS ON SUGAR METABOLISM

Reorganizations in the global metabolism, notably at the glycolytic level and aimed to enhance energy production seem to be crucial in the response of bifidobacteria and lactobacilli to bile. By increasing energy production, active responses against the detrimental action of bile at different levels, such as bile efflux, fatty acid biosynthesis and cell-wall architecture can be accomplished. However, particular metabolic shifts seem to be strain-dependent. Key enzymes of central metabolism such as phosphofructokinase, phosphoglycerate mutase, or elongation factor Tu were significantly over-expressed in response to bile salts in lactobacilli (Wu et al., 2010). Changes in the glycolytic metabolism, analyzed by measuring end-products, also pointed to a deep metabolic reorganization in lactobacilli as response to bile, suggesting an activation of central glycolysis (Lee et al., 2008; Burns et al., 2010). *B. animalis* and *B. longum* also demonstrated metabolic shifts in carbohydrate metabolism under bile environments, although the particular response appears to be strain-dependent. While *B. longum* accumulates most of the enzymes of the glycolytic pathway, suggesting an increase of glucose consumption in bile environments, *B. animalis* subsp. *lactis* displayed an accumulation of enzymes involved in the formation of fructose-6-phosphate, fructose-6-phosphate-phosphoketolase, and glyceraldehido-3-phosphate dehydrogenase, these being the only overproduced enzymes of the bifid shunt (Sánchez et al., 2005, 2007b). Physiological analysis confirmed an increased rate of glucose consumption in *B. longum* bile exposed cells but not in *B. animalis*. Therefore an increase in ATP production following bile challenge seems to occur through different routes: while *B. longum* increases ATP production through glycolysis, *B. animalis* increases the phosphorylation at substrate level (Sánchez et al., 2008).

Acquisition of bile tolerance was also associated to metabolic shifts in both bifidobacteria and lactobacilli strains. For instance, a bile-resistant *B. animalis* derivative, exhibited a maltose over glucose preference as compared to the parental strain, what might represent a selective advantage within the distal colon, where glucose is not available (Ruas-Madiedo et al., 2005). In addition, a proteomic comparison of bile response in both wild type and derivative strains, suggests that in the bile-adapted strain, the bifid shunt is displaced toward other metabolic pathways, i.e., oxalic degradation, that would theoretically increase ATP production (Sánchez et al., 2007b). Indeed, under bile environments, a bile-adapted *B. animalis* maintains a higher ATP concentration than its original counterpart (Sánchez et al., 2006). In *Lactobacillus*, bile adaptation resulted in higher glucose consumption and lactic acid formation, as compared to wild type strains (Burns et al., 2010). Interestingly, a bile-salt adapted strain of *L. delbrueckii* subsp. *lactis* decreased the production of ethanol through the glycolytic pathway, with a concomitant increase of aroma-related compounds, such as acetaldehyde, when grown in milk and with respect to the parental strain (Burns et al., 2011b). Therefore, care should be taken in the sense that bile-salt adaptation cannot only affect probiotic traits such adhesion (Burns et al., 2011a), but also important properties in food technology, such as variations in production of metabolic end products or resistance to bacteriophages (Guglielmotti et al., 2007; Burns et al., 2011b).

## CONCLUSION

Bile plays an important role in the physiology of intestinal bacteria, thus conditioning their functionality. This is particularly important for probiotic bacteria, since their beneficial effects must be generated in the presence of this biological fluid. In fact, we know that the activities of intestinal lactobacilli and bifidobacteria are deeply influenced by the presence of bile salts, and even some of them, such as cholesterol assimilation, have been directly correlated with bile salt metabolism in these bacteria. The understanding of the mechanisms by which probiotic bacteria are able to survive the stress caused by bile salts has remained elusive, but current -omics techniques have unraveled the protein and gene networks involved in this process, and delineated specific responses directed to cope with bile stress. It is remarkable that the existence of common mechanisms to cope with bile stress in probiotic bacteria belonging to phylogenetically different groups as is the case for *Bifidobacterium* and *Lactobacillus*, reflecting the existence of convergent evolutionary forces that have shaped probiotic tools to compete within the intestinal environment. Activation of molecular machinery to counteract oxidative and acid stresses are common responses to bile stress, as well as utilization of bile efflux systems and bile modification through bile salt hydrolases. Application of -omics methodologies to analyze strains performance *in vivo*, will undoubtedly shed valuable information to identify key players in which we can act in order to improve the survival of probiotics along the gastrointestinal tract.

## Conflict of Interest Statement

The authors declare that the research was conducted in the absence of any commercial or financial relationships that could be construed as a potential conflict of interest.
